# Allometric Coefficients for Body Measurements and Morphometric Indices in Young Huacaya Alpacas from the Peruvian Highlands

**DOI:** 10.3390/life15101529

**Published:** 2025-09-28

**Authors:** Ali William Canaza-Cayo, Roxana Churata-Huacani, Francisco Halley Rodriguez-Huanca, Diana Carla Fernandes Oliveira, Carola Trinidad Melo-Rojas, Rilke Tadeu Fonseca de Freitas, Luis Roque-Almanza, Maria Celeste Huanca-Ilaquijo, Edwin Amadeus Dueñas-Chaiña, Miguel del Carmen Rodriguez-Huanca, Júlio Sílvio de Sousa Bueno Filho

**Affiliations:** 1 Facultad de Ciencias Agrarias, Escuela Profesional de Ingeniería Agronómica, Universidad Nacional del Altiplano, Puno 21001, Peru; 2 Departamento de Estatística, Instituto de Ciências Exatas e Tecnológicas, Universidade Federal de Lavras, Lavras CEP 37200-900, MG, Brazil; jssbueno@ufla.br; 3 Departamento de Zootecnia, Universidade Federal de Lavras, Lavras CEP 37200-900, MG, Brazil; roxana.huacani2@estudante.ufla.br (R.C.-H.); diana.carla@ufla.br (D.C.F.O.); rilke@ufla.br (R.T.F.d.F.); 4 Facultad de Medicina Veterinária y Zootecnia, Universidad Nacional del Altiplano, Puno 21001, Peruhuancaceleste29@gmail.com (M.C.H.-I.); eduenaschaina@gmail.com (E.A.D.-C.); 5 Facultad de Ciencias Agrarias, Universidad Nacional San Antonio Abad del Cusco, Cusco 08001, Peru; carola.melo@unsaac.edu.pe (C.T.M.-R.); luis.roque@unsaac.edu.pe (L.R.-A.); 6 Facultad de Derecho, Universidad Continental, Arequipa 04016, Peru

**Keywords:** alpacas, morphometric indices, allometric growth, phenotypic characterization, sexual dimorphism

## Abstract

(1) Background: Alpacas play a crucial role in the livelihood and cultural heritage of Andean communities, yet limited scientific information exists regarding their morphometric growth patterns under high-altitude conditions. Understanding how environmental and biological factors influence their body development is essential for optimizing management and genetic improvement programs. (2) Methods: This study aimed to characterize the morphometric profile and allometric growth patterns of young Huacaya alpacas, evaluating the influence of sex, birth month, and fiber color on 18 linear body measurements and 6 morphometric indices from 146 animals. (3) Results: General linear models revealed that birth month had a significant effect (*p* < 0.05) on the compactness index, body side index, and body index while sex, fiber color, and their interaction did not significantly affect most indices. Allometric analysis showed that head traits exhibited low allometric coefficients (0.08–0.23), whereas torso-related measures such as dorsal length and abdominal perimeter showed higher coefficients (0.33 and 0.36, respectively). The compactness index showed marked sexual dimorphism in the allometric coefficient (0.83 in females, 0.95 in males). Thoracic perimeter exhibited a strong relationship with body weight and low variability, highlighting this measure as a key predictor of body size. (4) Morphometric and allometric analyses provide the first growth coefficients for young Huacaya alpacas at high altitude, offering a scientific basis for phenotypic selection of animals with superior meat potential and adaptability, thereby directly improving breeding efficiency and management in Andean production systems.

## 1. Introduction

Alpacas (*Vicugna pacos*), domesticated South American camelids, play a fundamental role in the economy of Andean highland communities, primarily due to the production of high-quality fiber [1,2]. However, in recent decades, there has been increasing interest in evaluating other productive traits, such as meat production, particularly in animals unsuitable for the textile industry [3,4]. Several studies have highlighted the nutritional value, technological quality, and market acceptance of alpaca meat in the Andean region [3,4,5]. The morphostructural characterization represents a valuable tool for identifying animals with greater productive potential, defining selection criteria, and optimizing breeding systems [6].

Morphometry, through the measurement of different body regions, enables the assessment of animal growth and body proportions. Its application in South American camelids has been more limited compared to other livestock species such as sheep and cattle [7,8]. Nevertheless, recent studies have demonstrated that morphometric indices can substantially contribute to the functional and productive characterization of these animals, allowing the identification of individuals with greater meat aptitude or adaptive resilience in harsh environments [9,10].

Allometric analysis, based on the study of relationships between body size and the growth of organs and tissues, has been widely used to model changes in body composition and carcass traits in livestock species [11,12,13]. By fitting the allometric function $y=ax^{b}$, it is possible to quantify the relative growth of body regions and classify it as isometric (proportional to live weight), positive or hyperallometric (accelerated), or negative or hypoallometric (decelerated). This approach was effectively applied by Sabbioni et al. [14] to describe ontogenetic development in Cornigliese sheep, identifying, for instance, hyperallometric growth in croup width and hypoallometric growth in cannon circumference. However, its application in alpacas remains scarce. This critical knowledge gap is particularly evident for young alpacas (crias) under high-altitude conditions, where rigorous morphometric and allometric references are lacking, limiting the development of precise breeding programs for this species.

The evaluation of body conformation through morphometry and allometry constitutes a key approach for inferring productive aptitude in ruminants, particularly in extensive production systems and local breeds where phenotypic records and breeding programs are often limited. This framework allows for the identification of traits of productive relevance and the establishment of selection criteria and objectives, which are valuable for guiding conservation and genetic improvement programs [14,15,16]. Therefore, the aim of the present study was to analyze morphometric growth in young Huacaya alpacas by evaluating body measurements, allometric coefficients, and morphometric indices, while considering the effects of sex, month of birth, and fiber color. This information is expected to contribute to the understanding of phenotypic variability in this species and provide a foundation for selection strategies aimed at improving productive performance.

## 2. Materials and Methods

### 2.1. Study Area, Animals and Experimental Design

Morphometric data were collected from a total of 146 young Huacaya alpacas aged 10 months (80 males and 66 females), born between January and March 2021, from the Quimsachata annex of the National Institute of Agrarian Innovation (INIA), Puno. The January–March birth window was selected as it represents the peak of the seasonal birthing period in the high-altitude Andes. This ensured age and environmental homogeneity across the sample, minimizing confounding effects from seasonal variability. Animals were randomly selected from the available population to ensure a representative sample and minimize selection bias. Only Huacaya alpacas were included due to their high availability at the study site, ensuring a sufficient sample size and avoiding morphological bias from breed differences. The station is located between the districts of Santa Lucía and Cabanillas (provinces of Lampa and San Román, Puno region), at 15°44′00″ S and 70°41′00″ W, at an average altitude of 4300 m a.s.l., approximately 118 km from the city of Puno. It covers an area of 6281.50 ha, of which 5849.94 ha (93.13%) consist of natural pastures that form the basis of alpaca feeding [17]. Average annual precipitation ranges from 400 to 688 mm, and the temperature varies from 3 °C (May–July) to 15 °C (September–December), with an annual mean of 7 °C [1]. All animals were managed under similar feeding and health conditions.

### 2.2. Morphometric Measurements

A set of morphometric traits was recorded using a biometric tape and a millimeter ruler, and conducted by two trained operators following standard procedures for small ruminants [18]. The measurements included head traits (head length, HL; ear length, EL; head width, HW; interorbital distance, ID; head height, HH), neck traits (neck length, NL; upper neck perimeter, UNP; lower neck perimeter, LNP), trunk traits (wither height, WH; back height, BH; rump height, RH; dorsal length, DL; distance between ischial tips, DBI; tail length, TL; thoracic perimeter, TP; abdominal perimeter, AP), limb traits (anterior fore-shank perimeter, AFP; hoof length, HoL), and body weight (BW), which was recorded with a portable electronic scale.

### 2.3. Morphometric Indexes

Based on the recorded measurements, several morphometric indexes were calculated to characterize body proportions, following the formulas commonly applied in small ruminants. These included the compactness index (CI = [BW × 100]/HW), body side index (BSI = [HW × 100]/DL), anamorphosis index (AI = [TP × TP]/WH), dactyl-thoracic index (DTI = [AFP × 100]/TP), body index (BI = [DL × 100]/TP), chest side index (ChSI = [WH × 100]/DL).

### 2.4. Statistical Analysis

All raw data were preliminarily tested for normality with Shapiro–Wilk test and confirmed to follow a normal distribution, after which descriptive statistics (mean, standard deviation, minimum and maximum) were estimated. General linear models (GLM) were then applied to evaluate the effect of fixed factors, such as sex, month of birth, fiber color, and the sex × color interaction, on each morphometric variable and index, using SAS software Version 9.4 [19]. The initial model included all possible interactions. Non-significant interactions (*p* > 0.05) were removed, retaining only the sex × fiber color interaction, ensuring a parsimonious and robust model. Thus, the final statistical model was defined as: $y_{ijkl}=\mu +s_{i}+b_{j}+f_{k}+{\left(sf\right)}_{ik}+\varepsilon_{ijkl}$, where $y_{ijkl}$ is the observed value of the dependent variable (a body measure or morphometric index), μ is the overall mean, $s_{i}$ the fixed effect of sex (male, female), $b_{j}$ the fixed effect of month of birth (January, February, March), $f_{k}$ the fixed effect of fiber color (black, brown, white), ${\left(sf\right)}_{ik}$ the interaction between sex and fiber color, and $\varepsilon_{ijkl}$ the residual error. For the significant sex × color interaction, simple effects analysis was performed using a BY group approach in SAS. Pairwise comparisons for fiber color within each sex were conducted with Tukey’s test, while comparisons for sex within each color relied on the F-test due to only two levels. Relative growth of body measures and morphometric indexes in relation to body weight (BW) was estimated by fitting the nonlinear allometric function $y=ax^{b}$, where y is the dependent variable (a body measurement, in cm, or a morphometric index), a the intercept, x the independent variable (BW, in kg), and b the allometric coefficient [20,21]. Comparisons of the equation parameters between sexes were also carried out using Tukey’s test.

## 3. Results

The study included a total of 146 Huacaya alpacas, of which 80 (54.8%) were males and 66 (45.2%) females. Animals were distributed according to month of birth as follows: January (33.6%), February (53.4%), and March (13.0%) (Table 1). This distribution enabled the evaluation of the effects of sex, fiber color, and month of birth on body measurements, morphometric indexes, and allometric coefficients.

The descriptive statistical analysis revealed a wide variability among the evaluated parameters (Table 2). Notably, traits such as head height (HH) and the distance between ischial tips (DBI) showed coefficients of variation (CV) exceeding 50%, indicating very high dispersion attributed to biological variability during early growth stages rather than measurement error and reflecting heterogeneous development rates in young alpacas. In contrast, thoracic perimeter (TP) and the anterior fore-shank perimeter (AFP) exhibited low variability (CV < 20%), whereas neck length (NL) presented moderate variability (CV = 21.8%). Among the morphometric indexes, the greatest heterogeneity was observed in the compactness index (CI) and, particularly, in the chest side index ChSI (CV > 45%), suggesting considerable structural diversity in body and thoracic conformation within the studied population.

The analysis of variance for body measurements is reported in Table 3. Sex did not show a statistically significant effect (*p* > 0.05) on any of the evaluated traits. In contrast, month of birth significantly affected several measurements, including head width (HW), head height (HH), abdominal perimeter (AP), lower neck perimeter (LNP), and hoof length (HoL). Fiber color did not have significant effects on morphometric traits (*p* > 0.05), showing only marginal influences on a few variables. The sex × color interaction was not significant for most traits, with the exception of hoof length (HoL).

Analyzing the morphometric indices (Table 4) showed that the month of birth had a statistically significant effect on the compactness index (CI), the body side index (BSI), and the body index (BI) (*p* < 0.05). In contrast, sex, fiber color, and the sex×color interaction did not show any significant effects on the evaluated indices (*p* > 0.05). The chest side index (ChSI) exhibited the highest variability, with a CV of 45.23%, whereas the body index (BI) showed the lowest variability (8.41%), suggesting greater phenotypic stability in the latter index.

The analysis of least squares means for the sex × color interaction (Table 5) revealed patterns of morphometric variation. Head height (HH) showed the most notable difference: black males had a substantially lower HH (70.38 cm) than females (88.32 cm), whereas the opposite pattern was observed in the white group, with males being taller (91.02 cm vs. 79.18 cm for females). White females recorded the highest abdominal perimeter (AP) (54.49 cm), while brown males showed the highest value within their group (54.19 cm). Other variables, such as neck length (NL) and dorsal length (DL), showed minor and less consistent differences among groups. These results reinforce previous evidence that fiber color appears to be associated with differences in the body conformation of Huacaya alpacas.

The analysis of least squares means for morphometric indices adjusted by the sex×color interaction (Table 6) showed specific trends. The compactness index (CI) was notably higher in black females (175.04) compared to males of the same color and other groups. The dactyl-thoracic index (DTI) was consistently higher in black and brown males; however, this trend was not maintained in the white group, where values were very similar between sexes. In contrast, the anamorphosis index (AI) proved to be the most stable, showing minimal variation among the different groups. Finally, the chest side index (ChSI) exhibited the greatest variability, with marked differences between colors and sexes. For instance, black females showed the highest value (79.57), whereas brown males showed the lowest (61.49). In the white group, males considerably surpassed females.

The allometric coefficients derived from the function $y=ax^{b}$ revealed distinct growth patterns across body regions (Table 7). Head measurements (HL, EL, HW, ID, HH) generally exhibited low coefficients (ranging from 0.00 to 0.39), albeit with considerable variation between sexes. For instance, HH showed a markedly higher coefficient in females (0.39) than in males (0.04), indicating early development but with sexual dimorphism. In contrast, torso and neck measurements such as WH, RH, DL, NL, TP, and AP displayed higher and more consistent coefficients (between 0.26 and 0.39), suggesting more prolonged and later growth. The DBI measurement showed a contrasting pattern, with a negative coefficient in males (−0.15) and a low positive coefficient in females (0.19), which may reflect functional differences in pelvic morphology. For limbs, AFP exhibited coefficients close to zero (females: −0.02, males: 0.05), suggesting very limited growth after weaning.

CI showed a notable difference between sexes, with a high positive allometric coefficient in males (0.95) and an even higher one in females (0.83). DTI showed negative values in both sexes (females: −0.36, males: −0.21), which was more pronounced in females, indicating a relative slowdown in the growth of the forearm perimeter relative to body weight. ChSI also displayed negative coefficients (females: −0.05, males: −0.18). In contrast, AI showed a moderate positive coefficient that was very similar between sexes (0.28), confirming its utility as a proportional descriptor of body development. Collectively, these results suggest that, despite the absence of a formal selection plan in the studied population, differentiated patterns of growth and body development exist between sexes, with the CI, DTI, and ChSI indices being particularly useful for characterizing productive aptitude in Huacaya alpacas.

## 4. Discussion

This study estimated the allometric coefficients of body measurements and morphometric indices in young Huacaya alpacas, aiming to evaluate the potential as indicators of growth and development for consideration in future selection programs. The results confirm extensive morphostructural variability in young Huacaya alpacas, consistent with that reported by Muñoz Barahona [22] and Mallma et al. [2] in herds reared in high-Andean systems. This structural heterogeneity was reflected in both linear measurements and morphometric indices, being more pronounced in variables related to the torso, such as abdominal perimeter (AP) and rump height (RH). The limited expression of sexual dimorphism at this early developmental stage, evidenced by the absence of a significant effect of the sex factor on morphometric variables, is consistent with observations by Ablondi et al. [23] in alpacas, Bacchi et al. [24] in guanacos, and Sabbioni et al. [14] in sheep. These authors note that sex differences are not significant when evaluating simple linear measurements during the rearing and yearling phases but emphasize that structural differences become more pronounced primarily in adulthood. On the other hand, the significant influence of the month of birth on characteristics such as abdominal perimeter and hoof length suggests that distinct seasonal environmental factors in the first months of life, possibly related to feed availability and rearing conditions, could have a greater impact on initial body development than sex or fiber color in this population. This aligns with Grund et al. [9], who found no growth differences by sex in young alpacas and attributed variations mainly to forage quality. These variations reflect the plasticity of body development in response to the environment, an aspect particularly relevant in high-altitude regions. Similar findings were reported by Buchallik-Schregel et al. [25], who showed that chest girth and withers height were strongly correlated with body condition and weight, highlighting external measurements as reliable indicators of nutritional status in alpacas.

The applicability of this study provides a scientific basis for phenotypic selection and management. Key findings, such as thoracic perimeter as a robust proxy for body weight and the allometric coefficients for growth patterns, offer simple, low-cost tools for producers to evaluate growth, meat potential, and body condition. This is particularly vital under high-altitude conditions where access to advanced technologies is limited. These tools directly inform breeding decisions, genetic improvement programs, and support the conservation of local alpaca populations.

Furthermore, the significant effect of the month of birth on the morphometric indices CI, BSI, and BI suggests that environmental factors associated with the time of birth, such as grazing availability, climatic conditions, or nutritional management in early life stages, could influence the animal’s proportional body development. These results agree with those reported by Sabbioni et al. [14], who emphasize that morphometric indices, especially those relating weight to body dimensions, are sensitive to variations in early growth, thus reflecting the impact of the environment on body conformation. Therefore, the month of birth could be considered not only an indicator of initial rearing conditions but also a useful variable for assessing harmonious development in management and selection programs. Comparable outcomes were observed by Ormachea et al. [26] in llamas, where prediction equations based on chest girth and body length proved highly reliable for estimating body weight under extensive systems.

The allometric coefficients indicate that the growth of body measurements in young Huacaya alpacas follows a differential pattern. Structures such as withers height (WH, 0.30) and body length (DL, 0.33) presented coefficients close to isometry, suggesting harmonious development of the axial skeleton. This pattern agrees with findings in sheep, where linear measurements show early growth associated with bone development [10]. The thoracic index (DTI, −0.28) showed a negative coefficient, indicating that thoracic perimeter increases more rapidly than height, a desirable trait in species with productive aptitude. This pattern suggests early maturation of the limbs relative to body mass, which may be adaptive for stability and locomotion in the rugged high-altitude terrain shortly after birth. Likewise, the high coefficient for abdominal perimeter (AP, 0.36) could reflect late growth of digestive capacity, similar to that observed in growing ruminants [25]. The anamorphosis index (AI, 0.27), considered an indicator of meat capacity, showed a relatively high and stable coefficient between sexes, suggesting that meat production in this population is a late-developing function, as described in meat sheep breeds [14]. These results indicate that morphometric indices are useful for evaluating the progress of body development in alpacas. Ablondi et al. [23] also demonstrated that incorporating morphometric indices substantially improved the accuracy of weight prediction models in alpacas, reinforcing their role as reliable growth indicators.

The allometric analysis provided key information on the differential growth rate in young Huacaya alpacas. Cranial measurements (HL, EL, HW) presented low allometric coefficients (between 0.08 and 0.23), suggesting early development of these structures, in line with patterns observed in ruminant species where the skull and its bony components mature before other body regions [14]. This pattern is consistent with the biological priority of developing sensory and feeding systems in early growth stages. In contrast, trunk measurements such as dorsal length (DL, 0.33), rump height (RH, 0.26), and especially abdominal perimeter (AP, 0.36) showed higher coefficients, indicating relatively later growth and greater plasticity associated with the development of the digestive system, as described in young camelids under extensive growth conditions [9]. This finding reinforces the idea that body development in alpacas follows a functional hierarchy, with skeletal structures maturing first and regions related to productive capacity developing later. The importance of these measurements as indicators of body development has been confirmed in studies on rabbits, where thoracic perimeter and body length showed significant allometric growth and high predictive capacity for live weight [27]. Similarly, Grund et al. [9] emphasized that chest girth is the most reliable predictor of body weight in alpacas, supporting its relevance as a key morphometric trait.

Some morphometric indices, such as the compactness index (CI) and the body side index (BSI), showed allometric coefficients differentiated between sexes, reflecting functionally distinct growth strategies. The CI, for example, presented higher values in males (0.95) than in females (0.83), which could indicate greater relative accumulation of body mass relative to height in males, a common pattern in species with incipient sexual dimorphism. This behavior is consistent with observations in meat sheep, where postnatal growth is prolonged longer in males than in females [14], and also in Brazilian native pigs, where males show positive allometric growth for body mass in relation to height [28]. The absence of significant differences by sex in most body measurements, as reported in Huacaya alpacas from other populations [29], suggests low sexual dimorphism in this species, at least during the first stages of life.

The absence of a formal selection program in the evaluated population likely contributes to the observed variability, as noted in studies on phenotypic diversity in camelids [23,25]. However, the significant effect of the month of birth on the compactness index (CI), body side index (BSI), and body index (BI) (*p* < 0.05), along with their coefficients of variation (20.5%, 13.5%, and 8.4%, respectively), suggests that early environmental factors-such as forage availability, climatic conditions, or nutritional management-influence the animal’s proportional development. This finding agrees with studies on guanacos, where morphological differences were strongly associated with food and water supply, indicating that ecological factors play a fundamental role in morphological development [24]. The identification of consistent structural patterns, such as the high correlation between thoracic perimeter (TP) and body weight, indicates that certain morphometric traits could be useful in phenotypic selection strategies. In this regard, Sabbioni et al. [14] emphasize that morphostructural characterization is particularly valuable in local breeds or populations without production records, as it allows inferring aptitudes and developmental trends from simple and non-invasive measurements.

Collectively, this study reinforces the utility of morphometry and allometry as accessible and efficient tools for characterizing body development in Huacaya alpacas, establishing a technical basis for improving phenotypic selection processes in herds raised under high-Andean conditions.

## 5. Conclusions

This study characterized the morphometrics traits of young Huacaya alpaca, revealing high phenotypic variability. No sexual dimorphism was detected in simple linear body measurements; however, allometric analysis indicated a greater relative body mass development in males. Birth month was the main environmental factor, significantly influencing body measurements and morphometric indices, highlighting the plasticity of growth in response to seasonal conditions. Morphometric indices (CI, DTI, ChSI) proved to be more sensitive than isolated measurements for assessing body conformation. Growth followed a sequential allometric pattern, with early development of cephalic structures and a prolonged growth of the trunk, associated with productive capacity. These findings confirm the utility of morphometric and allometric analyses as tools for phenotypic selection aimed at improving meat production in highland Huacaya alpacas. Furthermore, the morphometric benchmarks established in this study offer a critical resource for future research integrating artificial intelligence, such as computer vision, for non-invasive monitoring and management in alpaca production.

## Figures and Tables

**Table 1:** Number of young Huacaya alpacas and percentage (within brackets) of the subclasses of Sex × Month of birth involved in the study.

Month of Birth	Sex		Total
	Males	Females	
January	30 (20.5)	19 (13.0)	49 (33.6)
February	40 (27.4)	38 (26.0)	78 (53.4)
March	10 (6.8)	9 (6.2)	19 (13.0)
Total	80 (54.8)	66 (45.2)	146 (100)

**Table 2:** Descriptive statistics of parameters recorded from animals involved in the study.

Variable	Mean	SD	Maximum	Minimum
BW	19.8	3.3	29.0	13.5
Head measurements				
HL	20.6	2.8	27.0	1.5
EL	12.3	1.7	21.1	8.0
HW	11.9	1.9	17.0	7.0
ID	8.2	0.9	12.0	6.0
HH	78.8	43.2	125.0	10.0
Neck measurements				
NL	37.1	8.1	55.0	25.0
UNP	24.0	3.2	40.0	12.5
LNP	26.8	3.3	42.0	20.0
Torso Measurements				
WH	67.1	4.8	77.5	51.0
BH	46.4	21.2	76.5	21.5
RH	66.1	4.5	76.0	55.0
DL	55.5	4.7	69.0	42.1
DBI	8.2	4.3	55.0	5.0
TL	17.0	2.0	23.0	12.0
TP	66.8	4.7	81.0	52.0
AP	53.7	9.5	82.0	22.0
Limb measurements				
AFP	9.5	1.8	18.0	7.0
HoL	2.9	0.8	5.0	2.0
Morphometric indexes				
CI	169.8	35.3	263.8	92.0
BSI	21.4	2.9	28.5	14.3
AI	66.7	8.0	88.5	37.0
DTI	14.3	2.8	26.5	10.6
BI	83.4	7.2	113.8	64.8
ChSI	69.3	31.4	117.4	30.8

BW: Body Weight, HL: Head Length, EL: Ear Length, HW: Head Width, ID: Interorbital Distance, HH: Head Height, NL: Neck Length, UNP: Upper Neck Perimeter, LNP: Lower Neck Perimeter, WH: Wither Height, BH: Back Height, RH: Rump Height, DL: Dorsal Length, DBI: Distance Between Ischial Tips, TL: Tail Length, TP: Thoracic Perimeter, AP: Abdominal Perimeter, AFP: Anterior Fore-Shank Perimeter, HoL: Hoof Length, CI: Compactness Index, BSI: Body Side Index, AI: Anamorphosis Index, DTI: Dactyl-Thoracic Index, BI: Body Index, ChSI: Chest Side Index.

**Table 3:** Analysis of variance of body measures.

Traits	*p*-Value				CV (%)
	Sex	Month of Birth	Color	Sex × Color	
BW	0.268	0.099	0.865	0.789	16.841
Head measurements					
HL	0.273	0.089	0.531	0.124	13.193
EL	0.514	0.078	0.856	0.214	13.484
HW	0.748	0.008	0.602	0.987	15.639
ID	0.410	0.426	0.891	0.253	11.462
HH	0.541	0.001	0.168	0.285	52.284
Neck measurements					
NL	0.365	0.514	0.179	0.552	21.752
UNP	0.904	0.085	0.087	0.773	12.969
LNP	0.110	0.047	0.167	0.707	11.920
Torso Measurements					
WH	0.334	0.836	0.311	0.597	7.193
BH	0.982	0.594	0.314	0.160	45.701
RH	0.853	0.906	0.173	0.640	6.871
DL	0.321	0.470	0.731	0.953	8.524
DBI	0.352	0.332	0.671	0.667	53.481
TL	0.643	0.406	0.340	0.552	11.979
TP	0.939	0.115	0.846	0.735	7.131
AP	0.277	0.003	0.739	0.120	17.075
Limb measurements					
AFP	0.051	0.537	0.616	0.352	19.158
HoL	0.867	0.045	0.081	0.002	24.415

BW: Body Weight, HL: Head Length, EL: Ear Length, HW: Head Width, ID: Interorbital Distance, HH: Head Height, NL: Neck Length, UNP: Upper Neck Perimeter, LNP: Lower Neck Perimeter, WH: Wither Height, BH: Back Height, RH: Rump Height, DL: Dorsal Length, DBI: Distance Between Ischial Tips, TL: Tail Length, TP: Thoracic Perimeter, AP: Abdominal Perimeter, AFP: Anterior Fore-Shank Perimeter, HoL: Hoof Length.

**Table 4:** Analysis of variance of body indexes.

Traits	*p*-Value				CV (%)
	Sex	Month of Birth	Color	Sex × Color	
CI	0.547	0.007	0.970	0.867	20.528
BSI	0.757	0.031	0.483	0.995	13.537
AI	0.445	0.149	0.933	0.395	11.942
DTI	0.076	0.623	0.678	0.419	19.502
BI	0.287	0.019	0.859	0.572	8.406
ChSI	0.899	0.643	0.199	0.183	45.230

CI: Compactness Index, BSI: Body Side Index, AI: Anamorphosis Index, DTI: Dactyl-Thoracic Index, BI: Body Index, ChSI: Chest Side Index.

**Table 5:** Means of body weight (kg) and body measurements (cm) in young Huacaya alpacas as affected by sex × color interaction.

Trait	Black		Brown		White		RSE (%)
	Female	Male	Female	Male	Female	Male	
BW	20.64	19.72	20.24	19.37	19.68	19.52	1.00
HL	18.85	20.99	20.88	20.47	20.91	20.69	1.62
EL	12.81	12.11	12.32	11.92	12.06	12.55	1.12
HW	12.05	11.95	12.12	11.86	11.72	11.66	0.62
ID	8.08	8.28	8.31	8.13	8.05	8.44	0.76
HH	88.32	70.38	78.68	68.24	79.18	91.02	4.72
NL	34.88	35.75	39.08	37.80	38.12	34.82	2.03
UNP	25.54	25.01	23.23	23.50	23.93	24.44	1.50
LNP	25.85	26.24	26.04	27.60	26.88	27.65	1.21
WH	67.63	65.64	68.24	67.34	66.40	66.72	0.57
BH	53.33	46.48	45.27	41.33	42.82	52.60	4.33
RH	64.76	65.35	67.43	66.26	65.64	65.69	0.57
DL	55.60	54.63	56.56	55.37	55.54	54.97	0.49
DBI	7.75	7.62	7.79	9.39	7.43	8.28	3.65
TL	17.34	16.81	17.30	16.97	16.42	16.78	0.84
TP	66.73	66.74	66.47	67.35	67.08	66.37	0.23
AP	49.54	56.86	53.40	54.19	54.49	52.65	1.84
AFP	8.73	9.70	9.12	10.07	9.53	9.51	2.01
HoL	3.33	2.86	2.80	2.68	2.71	3.35	4.22

BW: Body Weight, HL: Head Length, EL: Ear Length, HW: Head Width, ID: Interorbital Distance, HH: Head Height, NL: Neck Length, UNP: Upper Neck Perimeter, LNP: Lower Neck Perimeter, WH: Wither Height, BH: Back Height, RH: Rump Height, DL: Dorsal Length, DBI: Distance Between Ischial Tips, TL: Tail Length, TP: Thoracic Perimeter, AP: Abdominal Perimeter, AFP: Anterior Fore-Shank Perimeter, HoL: Hoof Length.

**Table 6:** Means of body indexes in young Huacaya alpacas as affected by sex × color interaction.

Trait	Black		Brown		White		RSE (%)
	Female	Male	Female	Male	Female	Male	
CI	175.04	168.34	171.00	167.21	169.61	170.29	0.65
BSI	21.69	21.91	21.43	21.43	21.09	21.25	0.56
AI	65.95	68.39	65.11	67.66	67.95	66.29	0.79
DTI	13.08	14.54	13.76	14.98	14.40	14.37	1.92
BI	83.48	82.09	85.44	82.41	82.83	82.97	0.59
ChSI	79.57	70.70	66.25	61.49	64.36	79.19	4.46

CI: Compactness Index, BSI: Body Side Index, AI: Anamorphosis Index, DTI: Dactyl-Thoracic Index, BI: Body Index, ChSI: Chest Side Index.

**Table 7:** Intercepts (cm) of the allometric functions and allometric coefficients of body measures and body indexes on body weight in young Huacaya alpacas.

Traits	Intercept				Allometric Coefficient			
	Common	By Sex			Common	By Sex		
		Female	Male	*p*-Value		Female	Male	*p*-Value
Head measures								
HL	15.53	13.44	17.20	<0.0001	0.10	0.14	0.06	<0.0001
EL	6.11	5.44	6.72	<0.0001	0.23	0.27	0.20	<0.0001
HW	8.83	6.37	11.85	<0.0001	0.10	0.21	0.00	<0.0001
ID	6.59	7.68	5.76	<0.0001	0.08	0.02	0.12	<0.0001
HH	41.74	24.99	69.91	<0.0001	0.21	0.39	0.04	<0.0001
Neck measures								
NL	13.74	13.76	14.70	<0.0001	0.33	0.34	0.30	<0.0001
UNP	15.87	13.86	16.94	<0.0001	0.14	0.18	0.12	<0.0001
LNP	14.91	15.43	13.60	<0.0001	0.20	0.18	0.24	<0.0001
Torso measures								
WH	27.82	24.93	30.60	<0.0001	0.30	0.33	0.26	<0.0001
BH	32.24	24.80	38.77	<0.0001	0.12	0.21	0.06	<0.0001
RH	30.54	26.40	34.42	<0.0001	0.26	0.31	0.22	<0.0001
DL	20.80	18.69	23.02	<0.0001	0.33	0.37	0.29	<0.0001
DBI	9.37	4.33	13.44	<0.0001	−0.05	0.19	−0.15	<0.0001
TL	6.89	6.70	7.07	<0.0001	0.30	0.31	0.29	<0.0001
TP	28.51	26.16	29.92	<0.0001	0.29	0.31	0.27	<0.0001
AP	18.61	16.59	19.25	<0.0001	0.36	0.39	0.35	<0.0001
Limb measures								
AFP	9.43	9.73	8.47	<0.0001	0.00	−0.02	0.05	<0.0001
HoL	2.08	2.54	1.67	<0.0001	0.12	0.04	0.20	<0.0001
Body indexes								
AI	29.63	28.31	29.39	<0.0001	0.27	0.28	0.28	<0.0001
BI	73.21	69.90	78.71	<0.0001	0.04	0.06	0.02	<0.0001
BSI	42.17	33.24	51.05	<0.0001	−0.23	−0.15	−0.29	<0.0001
ChSI	11.89	78.77	118.46	<0.0001	0.89	−0.05	−0.18	<0.0001
CI	101.38	14.20	10.17	<0.0001	−0.13	0.83	0.95	<0.0001
DTI	33.25	40.34	27.35	<0.0001	−0.28	−0.36	−0.21	<0.0001

HL: Head Length, EL: Ear Length, HW: Head Width, ID: Interorbital Distance, HH: Head Height, NL: Neck Length, UNP: Upper Neck Perimeter, LNP: Lower Neck Perimeter, WH: Wither Height, BH: Back Height, RH: Rump Height, DL: Dorsal Length, DBI: Distance Between Ischial Tips, TL: Tail Length, TP: Thoracic Perimeter, AP: Abdominal Perimeter, AFP: Anterior Fore-Shank Perimeter, HoL: Hoof Length, CI: Compactness Index, BSI: Body Side Index, AI: Anamorphosis Index, DTI: Dactyl-Thoracic Index, BI: Body Index, ChSI: Chest Side Index.

## Data Availability

The authors will provide the raw data supporting the conclusions of this article upon request.
